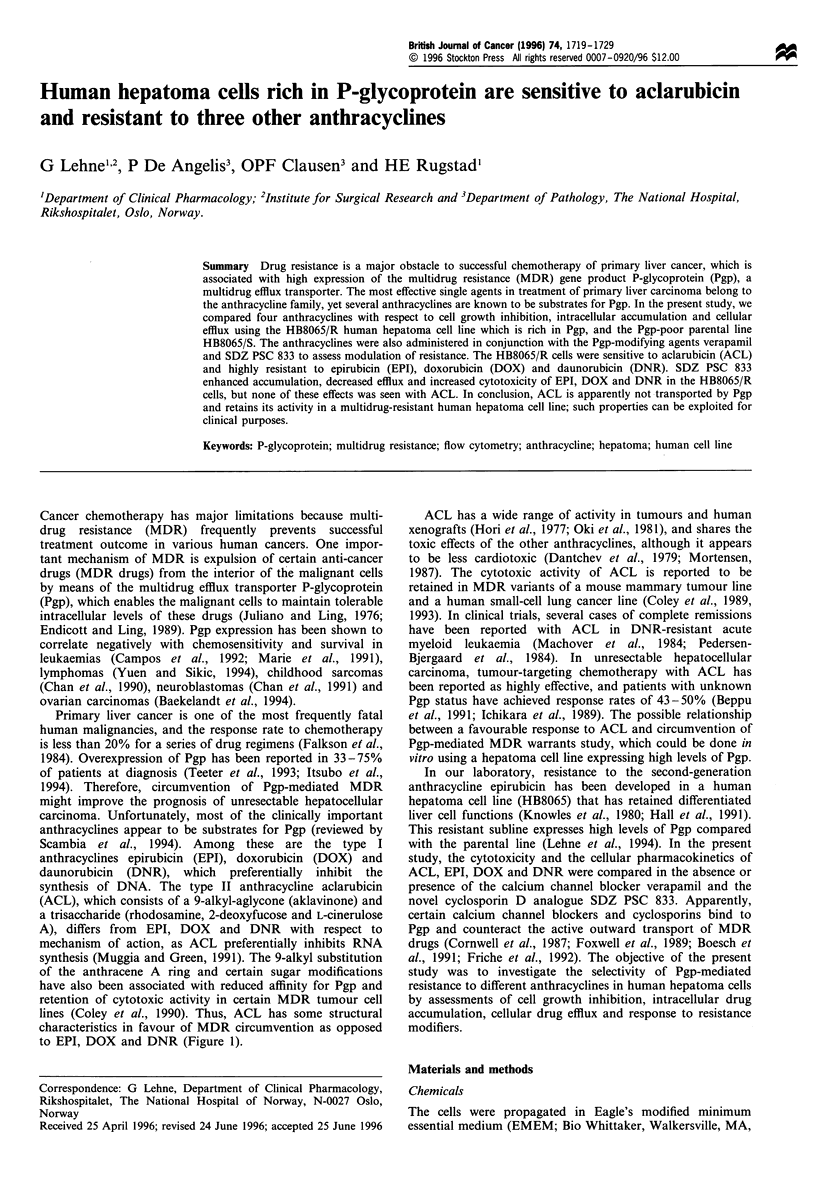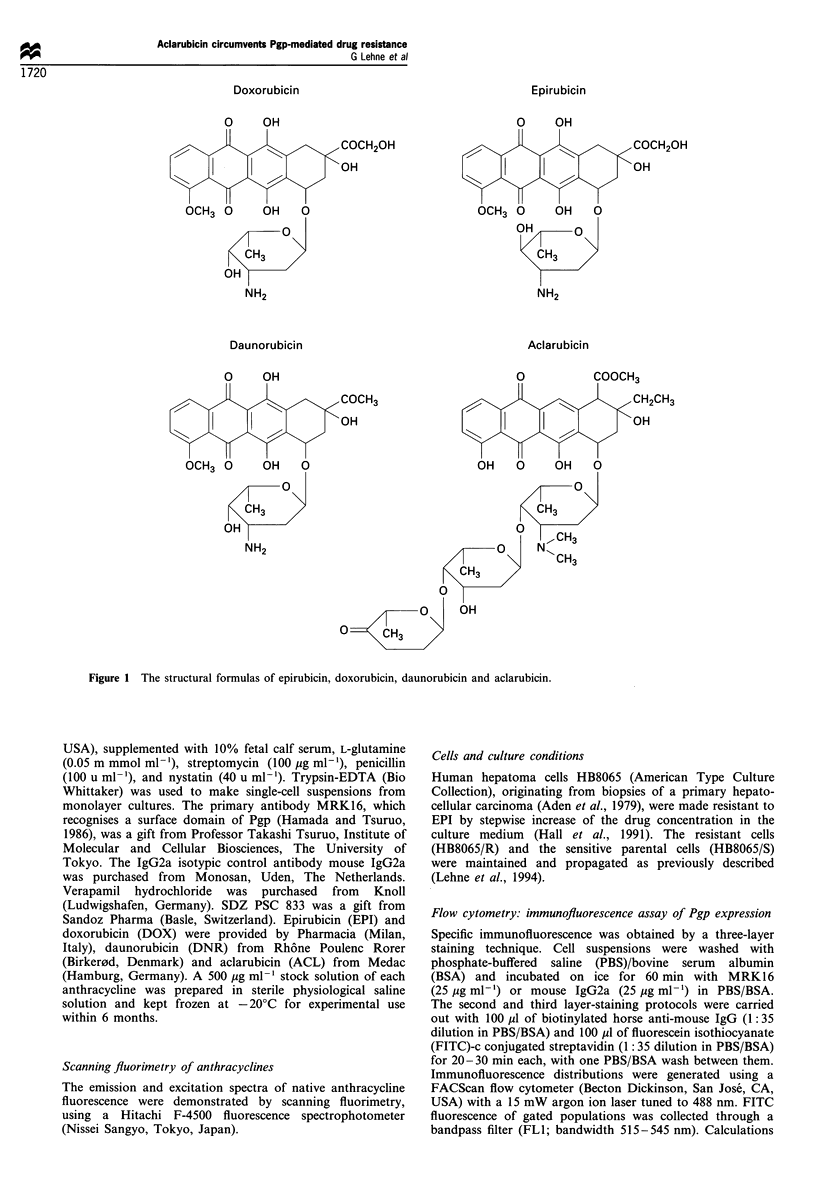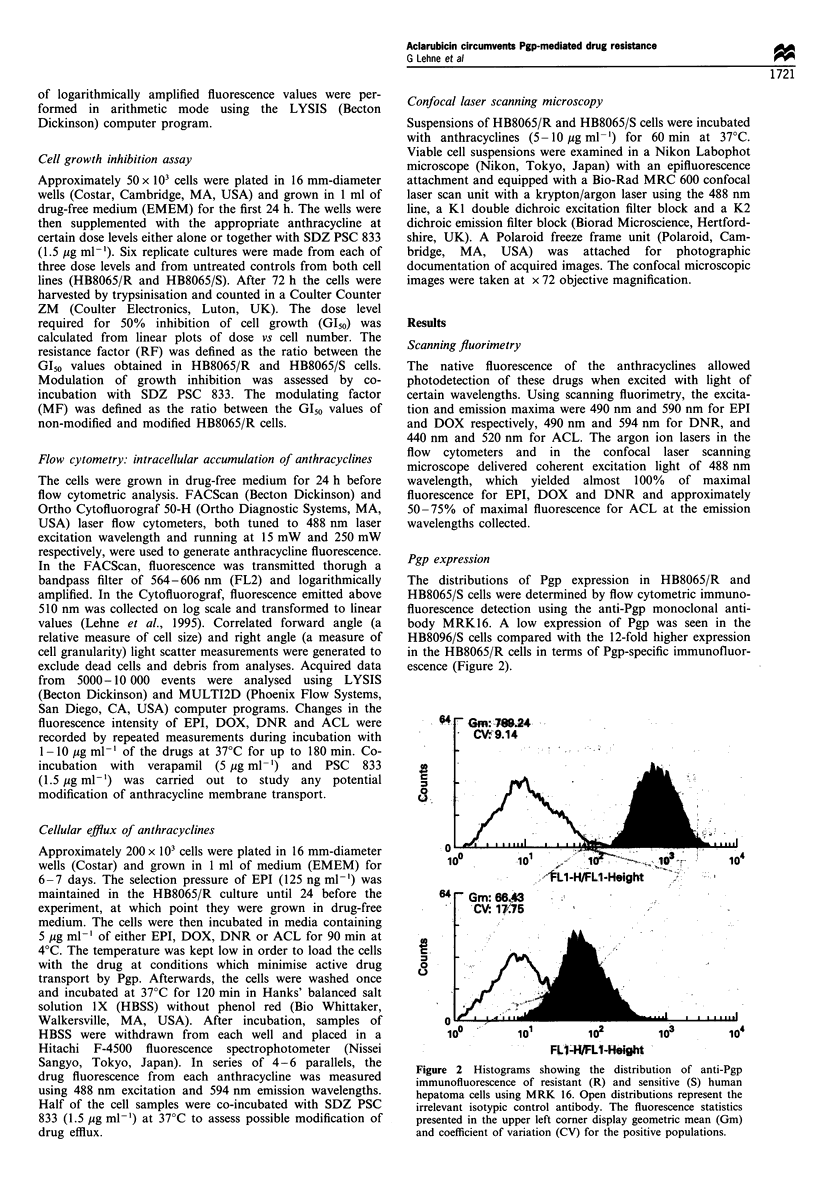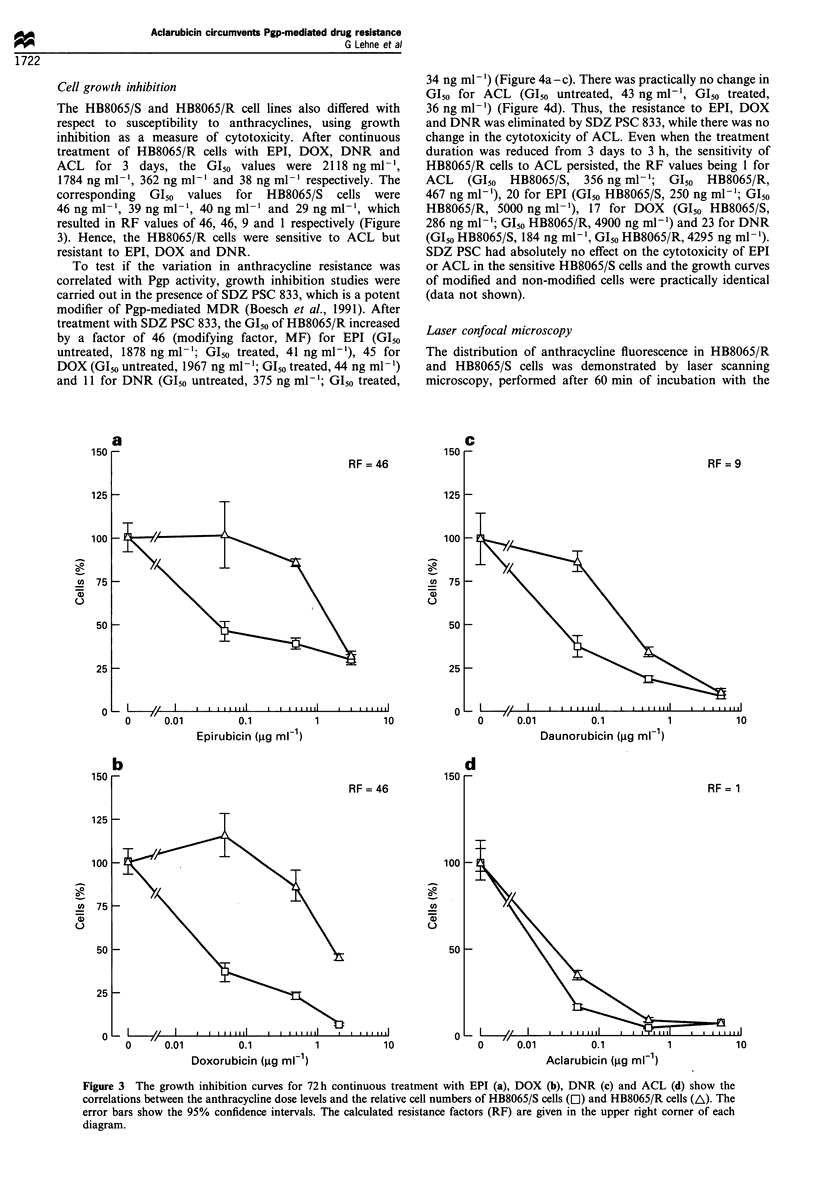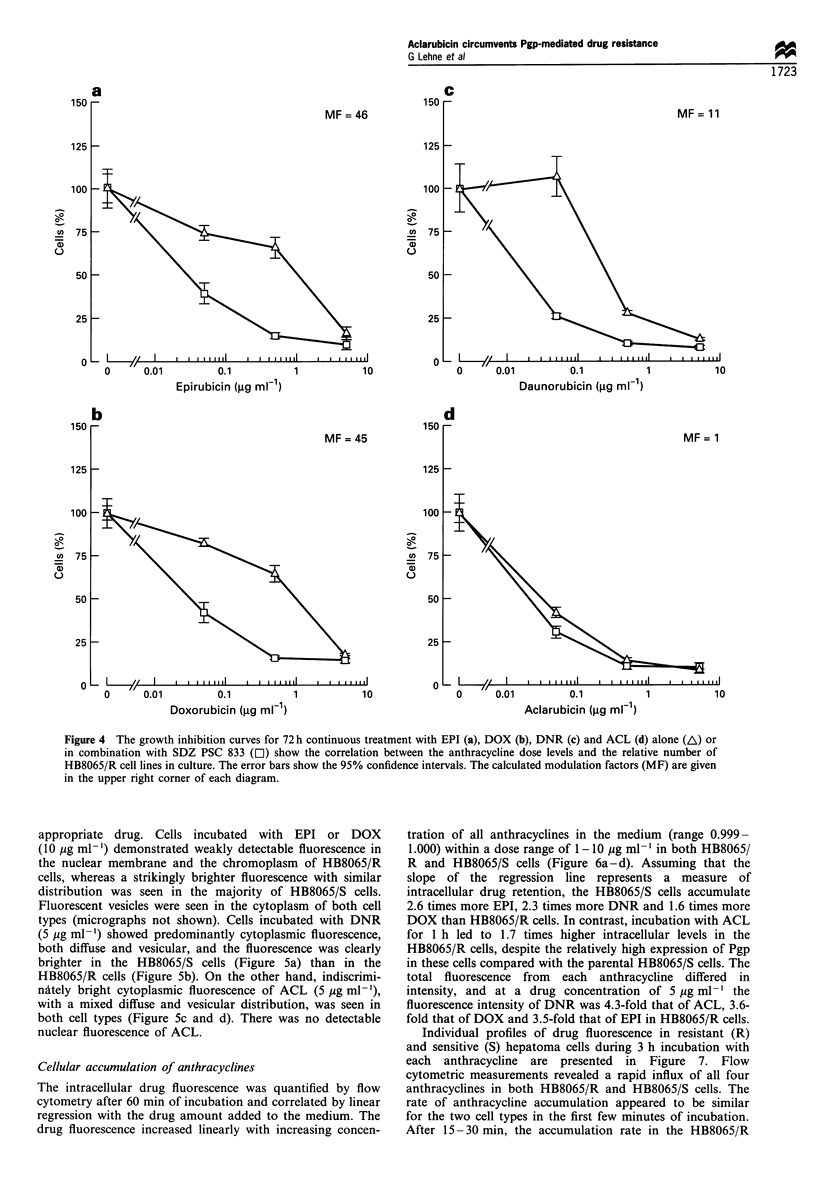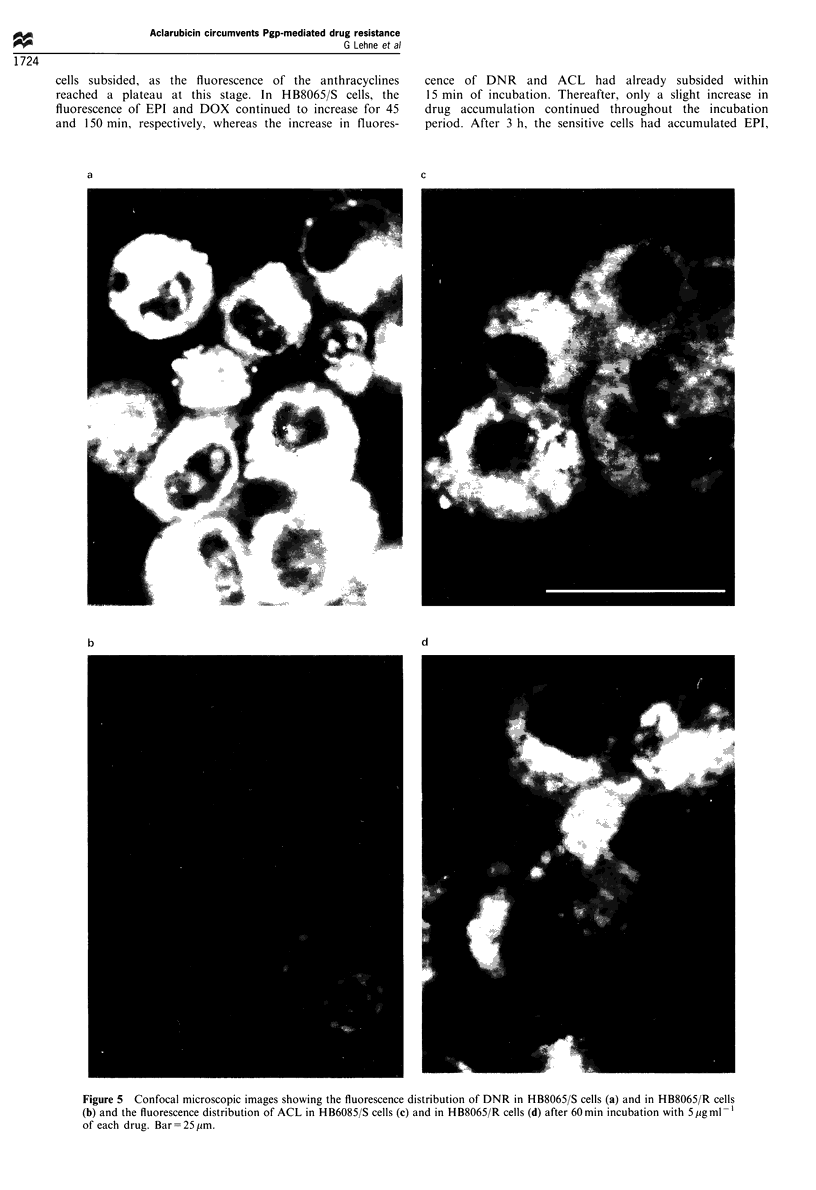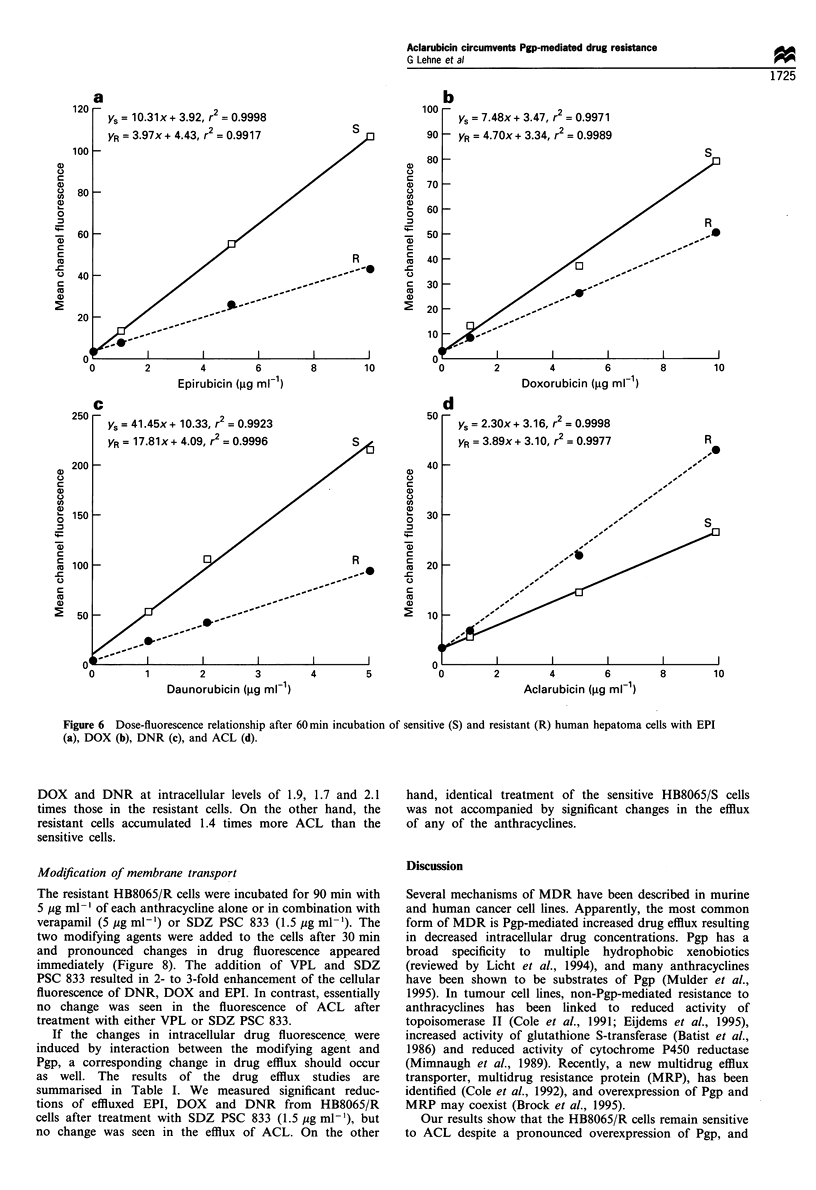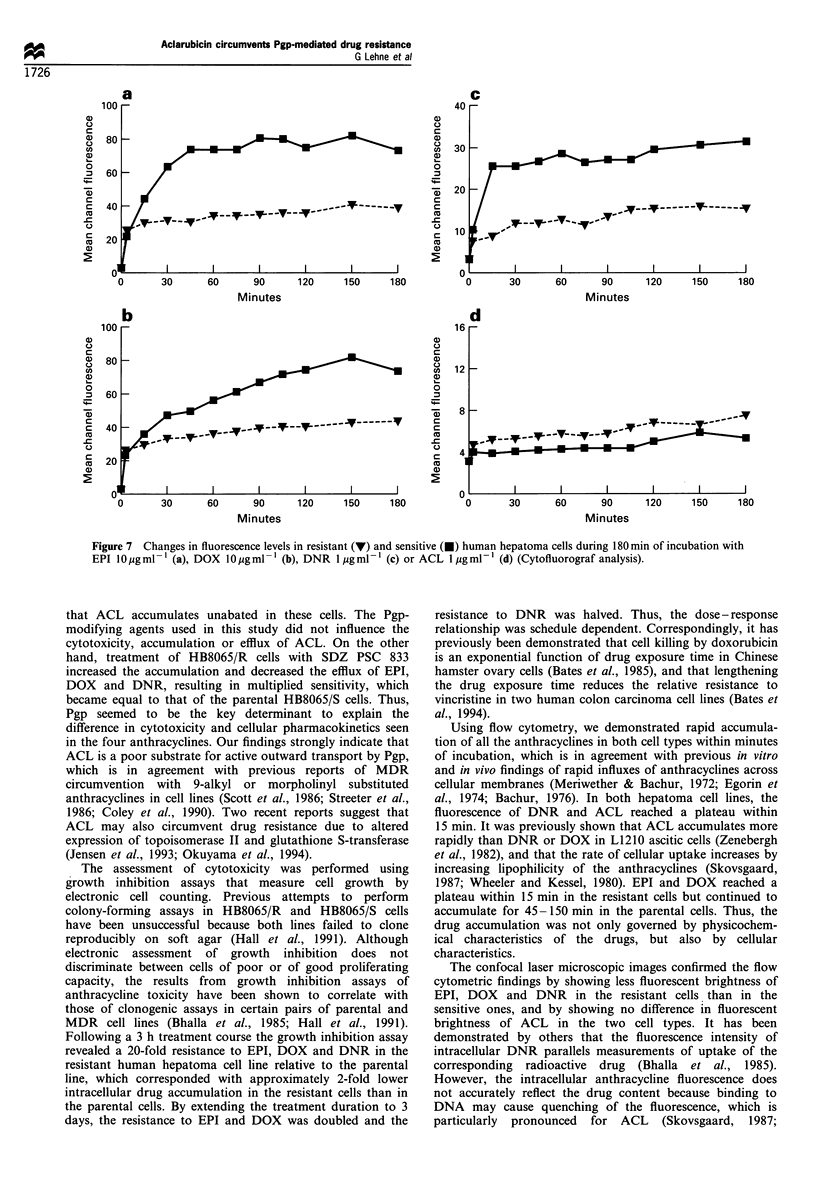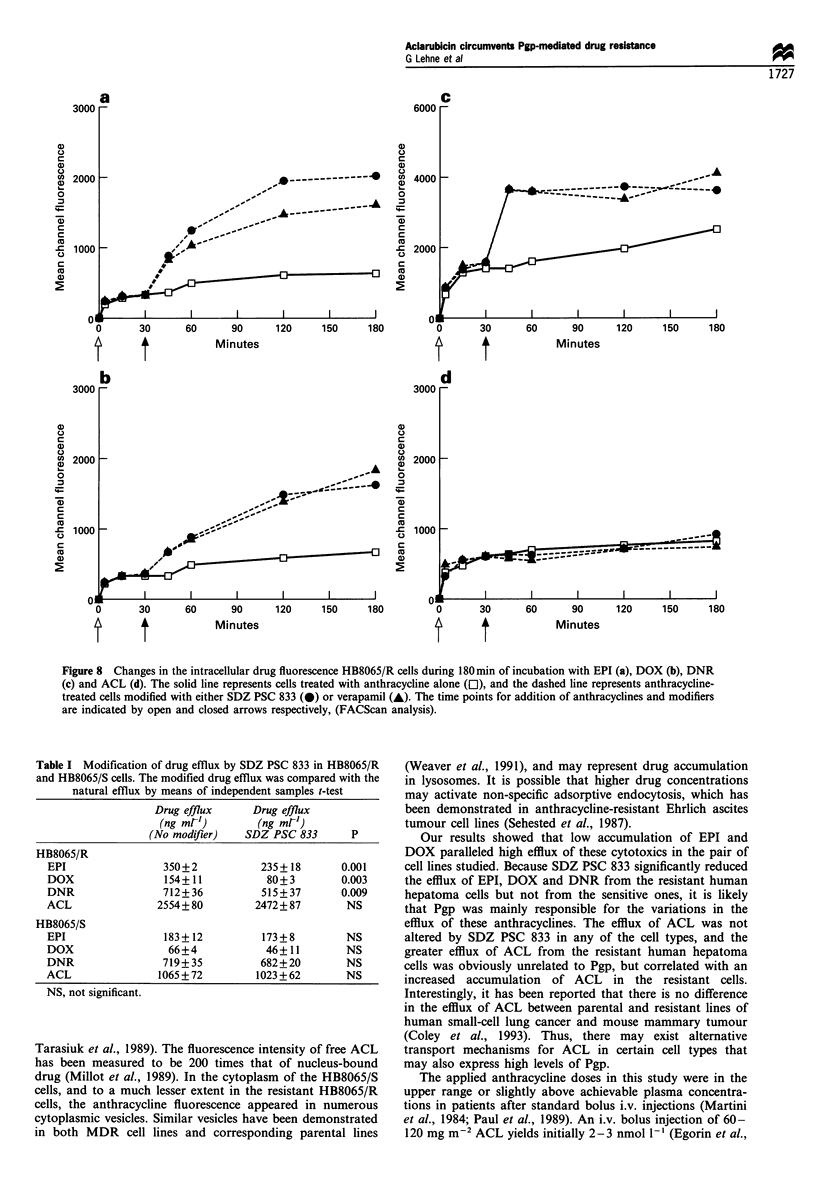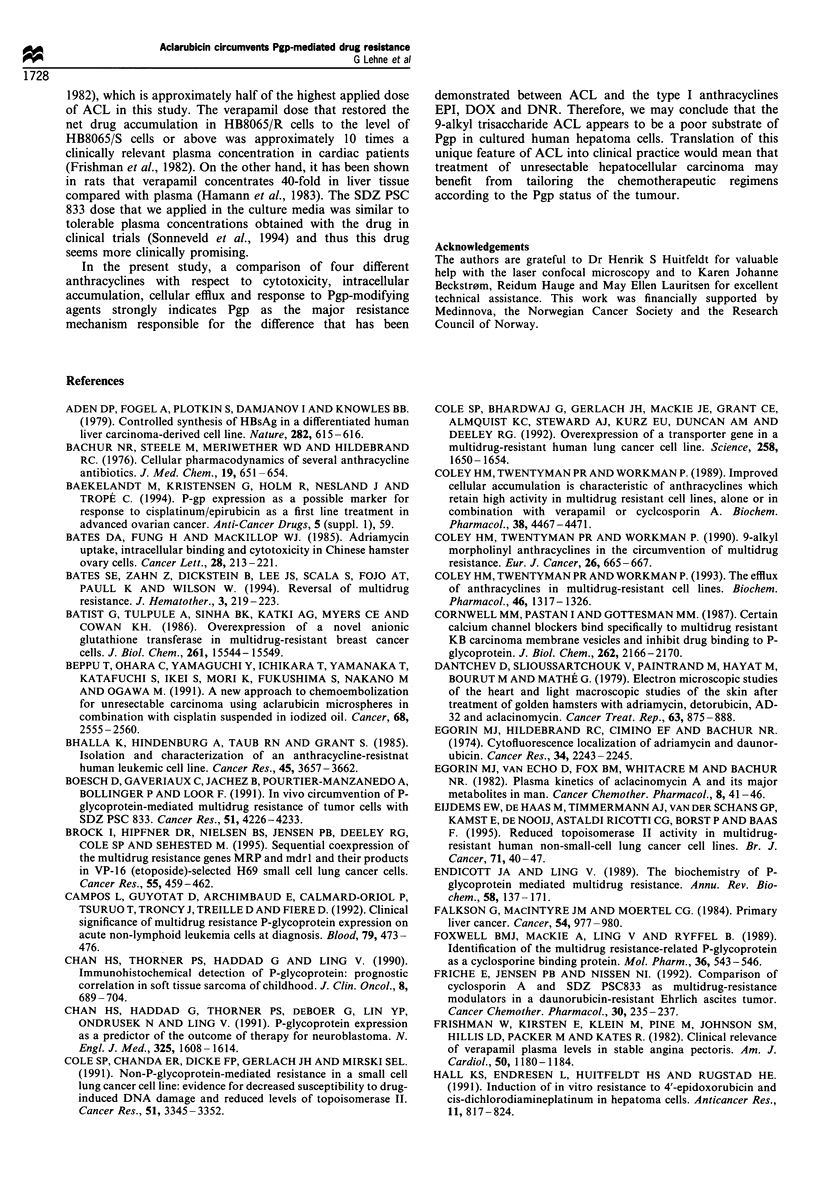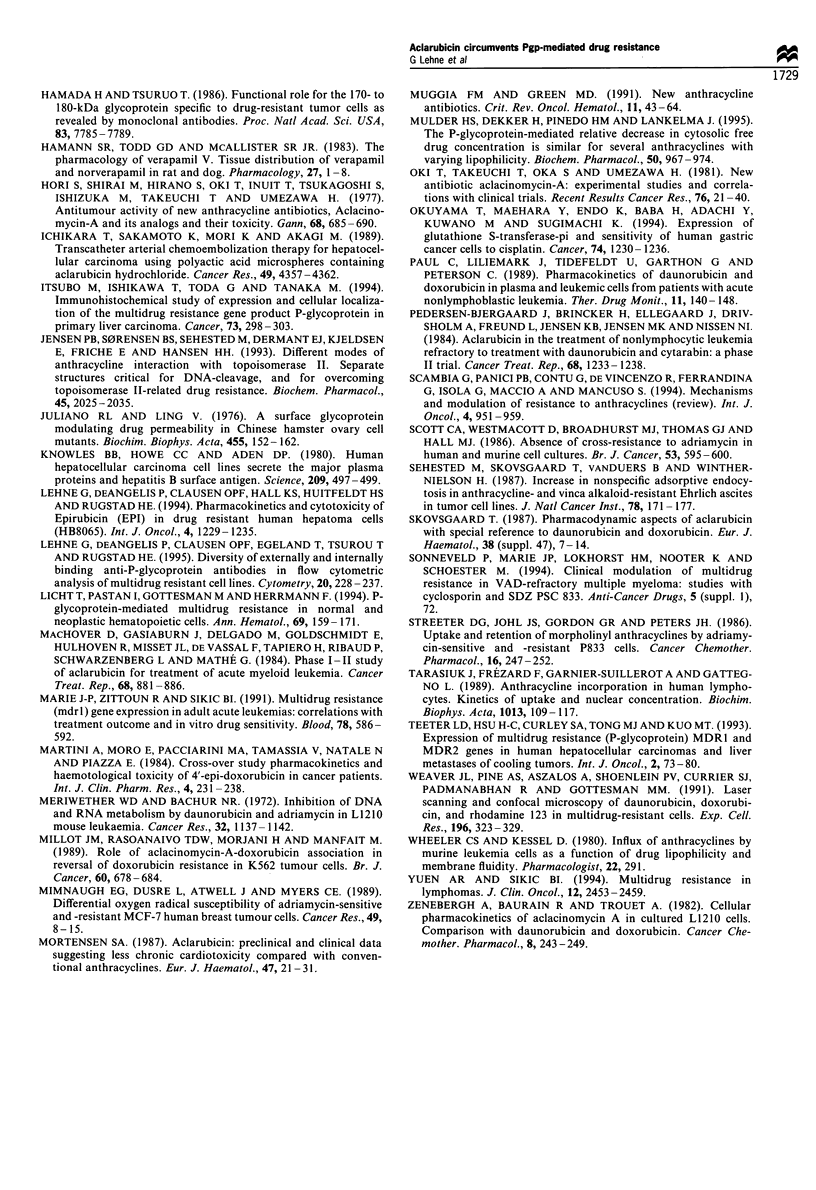# Human hepatoma cells rich in P-glycoprotein are sensitive to aclarubicin and resistant to three other anthracyclines.

**DOI:** 10.1038/bjc.1996.621

**Published:** 1996-12

**Authors:** G. Lehne, P. De Angelis, O. P. Clausen, H. E. Rugstad

**Affiliations:** Department of Clinical Pharmacology, National Hospital, Oslo, Norway.

## Abstract

**Images:**


British Journal of Cancer (1996) 74, 1719-1729
© 1996 Stockton Press All rights reserved 0007-0920/96 $12.00

### Human hepatoma cells rich in P-glycoprotein are sensitive to aclarubicin and resistant to three other anthracyclines

G Lehne[1,2], P De Angelis[3], OPF Clausen[3] and HE Rugstad[1]

[1]*Department of Clinical Pharmacology;* [2]*Institute for Surgical Research and* [3]*Department of Pathology, The National Hospital, Rikshospitalet, Oslo, Norway.*

**Summary** Drug resistance is a major obstacle to successful chemotherapy of primary liver cancer, which is associated with high expression of the multidrug resistance (MDR) gene product P-glycoprotein (Pgp), a multidrug efflux transporter. The most effective single agents in treatment of primary liver carcinoma belong to the anthracycline family, yet several anthracyclines are known to be substrates for Pgp. In the present study, we compared four anthracyclines with respect to cell growth inhibition, intracellular accumulation and cellular efflux using the HB8065/R human hepatoma cell line which is rich in Pgp, and the Pgp-poor parental line HB8065/S. The anthracyclines were also administered in conjunction with the Pgp-modifying agents verapamil and SDZ PSC 833 to assess modulation of resistance. The HB8065/R cells were sensitive to aclarubicin (ACL) and highly resistant to epirubicin (EPI), doxorubicin (DOX) and daunorubicin (DNR). SDZ PSC 833 enhanced accumulation, decreased efflux and increased cytotoxicity of EPI, DOX and DNR in the HB8065/R cells, but none of these effects was seen with ACL. In conclusion, ACL is apparently not transported by Pgp and retains its activity in a multidrug-resistant human hepatoma cell line; such properties can be exploited for clinical purposes.

**Keywords:** P-glycoprotein; multidrug resistance; flow cytometry; anthracycline; hepatoma; human cell line

Cancer chemotherapy has major limitations because multidrug resistance (MDR) frequently prevents successful treatment outcome in various human cancers. One important mechanism of MDR is expulsion of certain anti-cancer drugs (MDR drugs) from the interior of the malignant cells by means of the multidrug efflux transporter P-glycoprotein (Pgp), which enables the malignant cells to maintain tolerable intracellular levels of these drugs (Juliano and Ling, 1976; Endicott and Ling, 1989). Pgp expression has been shown to correlate negatively with chemosensitivity and survival in leukaemias (Campos *et al.*, 1992; Marie *et al.*, 1991), lymphomas (Yuen and Sikic, 1994), childhood sarcomas (Chan *et al.*, 1990), neuroblastomas (Chan *et al.*, 1991) and ovarian carcinomas (Baekelandt *et al.*, 1994).

Primary liver cancer is one of the most frequently fatal human malignancies, and the response rate to chemotherapy is less than 20% for a series of drug regimens (Falkson *et al.*, 1984). Overexpression of Pgp has been reported in 33–75% of patients at diagnosis (Teeter *et al.*, 1993; Itsubo *et al.*, 1994). Therefore, circumvention of Pgp-mediated MDR might improve the prognosis of unresectable hepatocellular carcinoma. Unfortunately, most of the clinically important anthracyclines appear to be substrates for Pgp (reviewed by Scambia *et al.*, 1994). Among these are the type I anthracyclines epirubicin (EPI), doxorubicin (DOX) and daunorubicin (DNR), which preferentially inhibit the synthesis of DNA. The type II anthracycline aclarubicin (ACL), which consists of a 9-alkyl-aglycone (aklavinone) and a trisaccharide (rhodosamine, 2-deoxyfucose and L-cinerulose A), differs from EPI, DOX and DNR with respect to mechanism of action, as ACL preferentially inhibits RNA synthesis (Muggia and Green, 1991). The 9-alkyl substitution of the anthracene A ring and certain sugar modifications have also been associated with reduced affinity for Pgp and retention of cytotoxic activity in certain MDR tumour cell lines (Coley *et al.*, 1990). Thus, ACL has some structural characteristics in favour of MDR circumvention as opposed to EPI, DOX and DNR (Figure 1).

Correspondence: G Lehne, Department of Clinical Pharmacology, Rikshospitalet, The National Hospital of Norway, N-0027 Oslo, Norway
Received 25 April 1996; revised 24 June 1996; accepted 25 June 1996

ACL has a wide range of activity in tumours and human xenografts (Hori *et al.*, 1977; Oki *et al.*, 1981), and shares the toxic effects of the other anthracyclines, although it appears to be less cardiotoxic (Dantchev *et al.*, 1979; Mortensen, 1987). The cytotoxic activity of ACL is reported to be retained in MDR variants of a mouse mammary tumour line and a human small-cell lung cancer line (Coley *et al.*, 1989, 1993). In clinical trials, several cases of complete remissions have been reported with ACL in DNR-resistant acute myeloid leukaemia (Machover *et al.*, 1984; Pedersen-Bjergaard *et al.*, 1984). In unresectable hepatocellular carcinoma, tumour-targeting chemotherapy with ACL has been reported as highly effective, and patients with unknown Pgp status have achieved response rates of 43–50% (Beppu *et al.*, 1991; Ichikara *et al.*, 1989). The possible relationship between a favourable response to ACL and circumvention of Pgp-mediated MDR warrants study, which could be done *in vitro* using a hepatoma cell line expressing high levels of Pgp.

In our laboratory, resistance to the second-generation anthracycline epirubicin has been developed in a human hepatoma cell line (HB8065) that has retained differentiated liver cell functions (Knowles *et al.*, 1980; Hall *et al.*, 1991). This resistant subline expresses high levels of Pgp compared with the parental line (Lehne *et al.*, 1994). In the present study, the cytotoxicity and the cellular pharmacokinetics of ACL, EPI, DOX and DNR were compared in the absence or presence of the calcium channel blocker verapamil and the novel cyclosporin D analogue SDZ PSC 833. Apparently, certain calcium channel blockers and cyclosporins bind to Pgp and counteract the active outward transport of MDR drugs (Cornwell *et al.*, 1987; Foxwell *et al.*, 1989; Boesch *et al.*, 1991; Friche *et al.*, 1992). The objective of the present study was to investigate the selectivity of Pgp-mediated resistance to different anthracyclines in human hepatoma cells by assessments of cell growth inhibition, intracellular drug accumulation, cellular drug efflux and response to resistance modifiers.

### Materials and methods

### *Chemicals*

The cells were propagated in Eagle's modified minimum essential medium (EMEM; Bio Whittaker, Walkersville, MA,

**Figure 1** The structural formulas of epirubicin, doxorubicin, daunorubicin and aclarubicin.

USA), supplemented with 10% fetal calf serum, L-glutamine (0.05 m mmol $ml^{-1}$), streptomycin (100 $\mu g$ $ml^{-1}$), penicillin (100 u $ml^{-1}$), and nystatin (40 u $ml^{-1}$). Trypsin-EDTA (Bio Whittaker) was used to make single-cell suspensions from monolayer cultures. The primary antibody MRK16, which recognises a surface domain of Pgp (Hamada and Tsuruo, 1986), was a gift from Professor Takashi Tsuruo, Institute of Molecular and Cellular Biosciences, The University of Tokyo. The IgG2a isotypic control antibody mouse IgG2a was purchased from Monosan, Uden, The Netherlands. Verapamil hydrochloride was purchased from Knoll (Ludwigshafen, Germany). SDZ PSC 833 was a gift from Sandoz Pharma (Basle, Switzerland). Epirubicin (EPI) and doxorubicin (DOX) were provided by Pharmacia (Milan, Italy), daunorubicin (DNR) from Rhône Poulenc Rorer (Birkerød, Denmark) and aclarubicin (ACL) from Medac (Hamburg, Germany). A 500 $\mu g$ $ml^{-1}$ stock solution of each anthracycline was prepared in sterile physiological saline solution and kept frozen at $-20°C$ for experimental use within 6 months.

*Scanning fluorimetry of anthracyclines*

The emission and excitation spectra of native anthracycline fluorescence were demonstrated by scanning fluorimetry, using a Hitachi F-4500 fluorescence spectrophotometer (Nissei Sangyo, Tokyo, Japan).

*Cells and culture conditions*

Human hepatoma cells HB8065 (American Type Culture Collection), originating from biopsies of a primary hepatocellular carcinoma (Aden *et al.*, 1979), were made resistant to EPI by stepwise increase of the drug concentration in the culture medium (Hall *et al.*, 1991). The resistant cells (HB8065/R) and the sensitive parental cells (HB8065/S) were maintained and propagated as previously described (Lehne *et al.*, 1994).

*Flow cytometry: immunofluorescence assay of Pgp expression*

Specific immunofluorescence was obtained by a three-layer staining technique. Cell suspensions were washed with phosphate-buffered saline (PBS)/bovine serum albumin (BSA) and incubated on ice for 60 min with MRK16 (25 $\mu g$ $ml^{-1}$) or mouse IgG2a (25 $\mu g$ $ml^{-1}$) in PBS/BSA. The second and third layer-staining protocols were carried out with 100 $\mu l$ of biotinylated horse anti-mouse IgG (1:35 dilution in PBS/BSA) and 100 $\mu l$ of fluorescein isothiocyanate (FITC)-c conjugated streptavidin (1:35 dilution in PBS/BSA) for 20–30 min each, with one PBS/BSA wash between them. Immunofluorescence distributions were generated using a FACScan flow cytometer (Becton Dickinson, San José, CA, USA) with a 15 mW argon ion laser tuned to 488 nm. FITC fluorescence of gated populations was collected through a bandpass filter (FL1; bandwidth 515–545 nm). Calculations of logarithmically amplified fluorescence values were performed in arithmetic mode using the LYSIS (Becton Dickinson) computer program.

*Cell growth inhibition assay*

Approximately $50 \times 10^3$ cells were plated in 16 mm-diameter wells (Costar, Cambridge, MA, USA) and grown in 1 ml of drug-free medium (EMEM) for the first 24 h. The wells were then supplemented with the appropriate anthracycline at certain dose levels either alone or together with SDZ PSC 833 (1.5 $\mu$g ml$^{-1}$). Six replicate cultures were made from each of three dose levels and from untreated controls from both cell lines (HB8065/R and HB8065/S). After 72 h the cells were harvested by trypsinisation and counted in a Coulter Counter ZM (Coulter Electronics, Luton, UK). The dose level required for 50% inhibition of cell growth ($GI_{50}$) was calculated from linear plots of dose *vs* cell number. The resistance factor (RF) was defined as the ratio between the $GI_{50}$ values obtained in HB8065/R and HB8065/S cells. Modulation of growth inhibition was assessed by co-incubation with SDZ PSC 833. The modulating factor (MF) was defined as the ratio between the $GI_{50}$ values of non-modified and modified HB8065/R cells.

*Flow cytometry: intracellular accumulation of anthracyclines*

The cells were grown in drug-free medium for 24 h before flow cytometric analysis. FACScan (Becton Dickinson) and Ortho Cytofluorograf 50-H (Ortho Diagnostic Systems, MA, USA) laser flow cytometers, both tuned to 488 nm laser excitation wavelength and running at 15 mW and 250 mW respectively, were used to generate anthracycline fluorescence. In the FACScan, fluorescence was transmitted thorugh a bandpass filter of 564–606 nm (FL2) and logarithmically amplified. In the Cytofluorograf, fluorescence emitted above 510 nm was collected on log scale and transformed to linear values (Lehne *et al.*, 1995). Correlated forward angle (a relative measure of cell size) and right angle (a measure of cell granularity) light scatter measurements were generated to exclude dead cells and debris from analyses. Acquired data from 5000–10 000 events were analysed using LYSIS (Becton Dickinson) and MULTI2D (Phoenix Flow Systems, San Diego, CA, USA) computer programs. Changes in the fluorescence intensity of EPI, DOX, DNR and ACL were recorded by repeated measurements during incubation with 1–10 $\mu$g ml$^{-1}$ of the drugs at 37°C for up to 180 min. Co-incubation with verapamil (5 $\mu$g ml$^{-1}$) and PSC 833 (1.5 $\mu$g ml$^{-1}$) was carried out to study any potential modification of anthracycline membrane transport.

*Cellular efflux of anthracyclines*

Approximately $200 \times 10^3$ cells were plated in 16 mm-diameter wells (Costar) and grown in 1 ml of medium (EMEM) for 6–7 days. The selection pressure of EPI (125 ng ml$^{-1}$) was maintained in the HB8065/R culture until 24 before the experiment, at which point they were grown in drug-free medium. The cells were then incubated in media containing 5 $\mu$g ml$^{-1}$ of either EPI, DOX, DNR or ACL for 90 min at 4°C. The temperature was kept low in order to load the cells with the drug at conditions which minimise active drug transport by Pgp. Afterwards, the cells were washed once and incubated at 37°C for 120 min in Hanks' balanced salt solution 1X (HBSS) without phenol red (Bio Whittaker, Walkersville, MA, USA). After incubation, samples of HBSS were withdrawn from each well and placed in a Hitachi F-4500 fluorescence spectrophotometer (Nissei Sangyo, Tokyo, Japan). In series of 4–6 parallels, the drug fluorescence from each anthracycline was measured using 488 nm excitation and 594 nm emission wavelengths. Half of the cell samples were co-incubated with SDZ PSC 833 (1.5 $\mu$g ml$^{-1}$) at 37°C to assess possible modification of drug efflux.

*Confocal laser scanning microscopy*

Suspensions of HB8065/R and HB8065/S cells were incubated with anthracyclines (5–10 $\mu$g ml$^{-1}$) for 60 min at 37°C. Viable cell suspensions were examined in a Nikon Labophot microscope (Nikon, Tokyo, Japan) with an epifluorescence attachment and equipped with a Bio-Rad MRC 600 confocal laser scan unit with a krypton/argon laser using the 488 nm line, a K1 double dichroic excitation filter block and a K2 dichroic emission filter block (Biorad Microscience, Hertfordshire, UK). A Polaroid freeze frame unit (Polaroid, Cambridge, MA, USA) was attached for photographic documentation of acquired images. The confocal microscopic images were taken at ×72 objective magnification.

### Results

*Scanning fluorimetry*

The native fluorescence of the anthracyclines allowed photodetection of these drugs when excited with light of certain wavelengths. Using scanning fluorimetry, the excitation and emission maxima were 490 nm and 590 nm for EPI and DOX respectively, 490 nm and 594 nm for DNR, and 440 nm and 520 nm for ACL. The argon ion lasers in the flow cytometers and in the confocal laser scanning microscope delivered coherent excitation light of 488 nm wavelength, which yielded almost 100% of maximal fluorescence for EPI, DOX and DNR and approximately 50–75% of maximal fluorescence for ACL at the emission wavelengths collected.

*Pgp expression*

The distributions of Pgp expression in HB8065/R and HB8065/S cells were determined by flow cytometric immunofluorescence detection using the anti-Pgp monoclonal antibody MRK16. A low expression of Pgp was seen in the HB8096/S cells compared with the 12-fold higher expression in the HB8065/R cells in terms of Pgp-specific immunofluorescence (Figure 2).

**Figure 2** Histograms showing the distribution of anti-Pgp immunofluorescence of resistant (R) and sensitive (S) human hepatoma cells using MRK 16. Open distributions represent the irrelevant isotypic control antibody. The fluorescence statistics presented in the upper left corner display geometric mean (Gm) and coefficient of variation (CV) for the positive populations.

*Cell growth inhibition*

The HB8065/S and HB8065/R cell lines also differed with respect to susceptibility to anthracyclines, using growth inhibition as a measure of cytotoxicity. After continuous treatment of HB8065/R cells with EPI, DOX, DNR and ACL for 3 days, the $GI_{50}$ values were 2118 ng $ml^{-1}$, 1784 ng $ml^{-1}$, 362 ng $ml^{-1}$ and 38 ng $ml^{-1}$ respectively. The corresponding $GI_{50}$ values for HB8065/S cells were 46 ng $ml^{-1}$, 39 ng $ml^{-1}$, 40 ng $ml^{-1}$ and 29 ng $ml^{-1}$, which resulted in RF values of 46, 46, 9 and 1 respectively (Figure 3). Hence, the HB8065/R cells were sensitive to ACL but resistant to EPI, DOX and DNR.

To test if the variation in anthracycline resistance was correlated with Pgp activity, growth inhibition studies were carried out in the presence of SDZ PSC 833, which is a potent modifier of Pgp-mediated MDR (Boesch *et al.*, 1991). After treatment with SDZ PSC 833, the $GI_{50}$ of HB8065/R increased by a factor of 46 (modifying factor, MF) for EPI ($GI_{50}$ untreated, 1878 ng $ml^{-1}$; $GI_{50}$ treated, 41 ng $ml^{-1}$), 45 for DOX ($GI_{50}$ untreated, 1967 ng $ml^{-1}$; $GI_{50}$ treated, 44 ng $ml^{-1}$) and 11 for DNR ($GI_{50}$ untreated, 375 ng $ml^{-1}$; $GI_{50}$ treated, 34 ng $ml^{-1}$) (Figure 4a–c). There was practically no change in $GI_{50}$ for ACL ($GI_{50}$ untreated, 43 ng $ml^{-1}$, $GI_{50}$ treated, 36 ng $ml^{-1}$) (Figure 4d). Thus, the resistance to EPI, DOX and DNR was eliminated by SDZ PSC 833, while there was no change in the cytotoxicity of ACL. Even when the treatment duration was reduced from 3 days to 3 h, the sensitivity of HB8065/R cells to ACL persisted, the RF values being 1 for ACL ($GI_{50}$ HB8065/S, 356 ng $ml^{-1}$; $GI_{50}$ HB8065/R, 467 ng $ml^{-1}$), 20 for EPI ($GI_{50}$ HB8065/S, 250 ng $ml^{-1}$; $GI_{50}$ HB8065/R, 5000 ng $ml^{-1}$), 17 for DOX ($GI_{50}$ HB8065/S, 286 ng $ml^{-1}$; $GI_{50}$ HB8065/R, 4900 ng $ml^{-1}$) and 23 for DNR ($GI_{50}$ HB8065/S, 184 ng $ml^{-1}$, $GI_{50}$ HB8065/R, 4295 ng $ml^{-1}$). SDZ PSC had absolutely no effect on the cytotoxicity of EPI or ACL in the sensitive HB8065/S cells and the growth curves of modified and non-modified cells were practically identical (data not shown).

*Laser confocal microscopy*

The distribution of anthracycline fluorescence in HB8065/R and HB8065/S cells was demonstrated by laser scanning microscopy, performed after 60 min of incubation with the

**Figure 3** The growth inhibition curves for 72 h continuous treatment with EPI (**a**), DOX (**b**), DNR (**c**) and ACL (**d**) show the correlations between the anthracycline dose levels and the relative cell numbers of HB8065/S cells (□) and HB8065/R cells (△). The error bars show the 95% confidence intervals. The calculated resistance factors (RF) are given in the upper right corner of each diagram.

**Figure 4** The growth inhibition curves for 72 h continuous treatment with EPI (**a**), DOX (**b**), DNR (**c**) and ACL (**d**) alone (△) or in combination with SDZ PSC 833 (□) show the correlation between the anthracycline dose levels and the relative number of HB8065/R cell lines in culture. The error bars show the 95% confidence intervals. The calculated modulation factors (MF) are given in the upper right corner of each diagram.

appropriate drug. Cells incubated with EPI or DOX (10 $\mu$g ml$^{-1}$) demonstrated weakly detectable fluorescence in the nuclear membrane and the chromoplasm of HB8065/R cells, whereas a strikingly brighter fluorescence with similar distribution was seen in the majority of HB8065/S cells. Fluorescent vesicles were seen in the cytoplasm of both cell types (micrographs not shown). Cells incubated with DNR (5 $\mu$g ml$^{-1}$) showed predominantly cytoplasmic fluorescence, both diffuse and vesicular, and the fluorescence was clearly brighter in the HB8065/S cells (Figure 5a) than in the HB8065/R cells (Figure 5b). On the other hand, indiscriminàtely bright cytoplasmic fluorescence of ACL (5 $\mu$g ml$^{-1}$), with a mixed diffuse and vesicular distribution, was seen in both cell types (Figure 5c and d). There was no detectable nuclear fluorescence of ACL.

### *Cellular accumulation of anthracyclines*

The intracellular drug fluorescence was quantified by flow cytometry after 60 min of incubation and correlated by linear regression with the drug amount added to the medium. The drug fluorescence increased linearly with increasing concentration of all anthracyclines in the medium (range 0.999–1.000) within a dose range of 1–10 $\mu$g ml$^{-1}$ in both HB8065/R and HB8065/S cells (Figure 6a–d). Assuming that the slope of the regression line represents a measure of intracellular drug retention, the HB8065/S cells accumulate 2.6 times more EPI, 2.3 times more DNR and 1.6 times more DOX than HB8065/R cells. In contrast, incubation with ACL for 1 h led to 1.7 times higher intracellular levels in the HB8065/R cells, despite the relatively high expression of Pgp in these cells compared with the parental HB8065/S cells. The total fluorescence from each anthracycline differed in intensity, and at a drug concentration of 5 $\mu$g ml$^{-1}$ the fluorescence intensity of DNR was 4.3-fold that of ACL, 3.6-fold that of DOX and 3.5-fold that of EPI in HB8065/R cells.

Individual profiles of drug fluorescence in resistant (R) and sensitive (S) hepatoma cells during 3 h incubation with each anthracycline are presented in Figure 7. Flow cytometric measurements revealed a rapid influx of all four anthracyclines in both HB8065/R and HB8065/S cells. The rate of anthracycline accumulation appeared to be similar for the two cell types in the first few minutes of incubation. After 15–30 min, the accumulation rate in the HB8065/R

cells subsided, as the fluorescence of the anthracyclines reached a plateau at this stage. In HB8065/S cells, the fluorescence of EPI and DOX continued to increase for 45 and 150 min, respectively, whereas the increase in fluorescence of DNR and ACL had already subsided within 15 min of incubation. Thereafter, only a slight increase in drug accumulation continued throughout the incubation period. After 3 h, the sensitive cells had accumulated EPI,

**Figure 5** Confocal microscopic images showing the fluorescence distribution of DNR in HB8065/S cells (**a**) and in HB8065/R cells (**b**) and the fluorescence distribution of ACL in HB6085/S cells (**c**) and in HB8065/R cells (**d**) after 60 min incubation with 5 $\mu g\,ml^{-1}$ of each drug. Bar = 25 $\mu m$.

**Figure 6** Dose-fluorescence relationship after 60 min incubation of sensitive (S) and resistant (R) human hepatoma cells with EPI (**a**), DOX (**b**), DNR (**c**), and ACL (**d**).

DOX and DNR at intracellular levels of 1.9, 1.7 and 2.1 times those in the resistant cells. On the other hand, the resistant cells accumulated 1.4 times more ACL than the sensitive cells.

*Modification of membrane transport*

The resistant HB8065/R cells were incubated for 90 min with 5 $\mu$g ml$^{-1}$ of each anthracycline alone or in combination with verapamil (5 $\mu$g ml$^{-1}$) or SDZ PSC 833 (1.5 $\mu$g ml$^{-1}$). The two modifying agents were added to the cells after 30 min and pronounced changes in drug fluorescence appeared immediately (Figure 8). The addition of VPL and SDZ PSC 833 resulted in 2- to 3-fold enhancement of the cellular fluorescence of DNR, DOX and EPI. In contrast, essentially no change was seen in the fluorescence of ACL after treatment with either VPL or SDZ PSC 833.

If the changes in intracellular drug fluorescence were induced by interaction between the modifying agent and Pgp, a corresponding change in drug efflux should occur as well. The results of the drug efflux studies are summarised in Table I. We measured significant reductions of effluxed EPI, DOX and DNR from HB8065/R cells after treatment with SDZ PSC 833 (1.5 $\mu$g ml$^{-1}$), but no change was seen in the efflux of ACL. On the other hand, identical treatment of the sensitive HB8065/S cells was not accompanied by significant changes in the efflux of any of the anthracyclines.

### Discussion

Several mechanisms of MDR have been described in murine and human cancer cell lines. Apparently, the most common form of MDR is Pgp-mediated increased drug efflux resulting in decreased intracellular drug concentrations. Pgp has a broad specificity to multiple hydrophobic xenobiotics (reviewed by Licht *et al.*, 1994), and many anthracyclines have been shown to be substrates of Pgp (Mulder *et al.*, 1995). In tumour cell lines, non-Pgp-mediated resistance to anthracyclines has been linked to reduced activity of topoisomerase II (Cole *et al.*, 1991; Eijdems *et al.*, 1995), increased activity of glutathione S-transferase (Batist *et al.*, 1986) and reduced activity of cytochrome P450 reductase (Mimnaugh *et al.*, 1989). Recently, a new multidrug efflux transporter, multidrug resistance protein (MRP), has been identified (Cole *et al.*, 1992), and overexpression of Pgp and MRP may coexist (Brock *et al.*, 1995).

Our results show that the HB8065/R cells remain sensitive to ACL despite a pronounced overexpression of Pgp, and

**Figure 7** Changes in fluorescence levels in resistant (▼) and sensitive (■) human hepatoma cells during 180 min of incubation with EPI 10 $\mu$g ml$^{-1}$ (**a**), DOX 10 $\mu$g ml$^{-1}$ (**b**), DNR 1 $\mu$g ml$^{-1}$ (**c**) or ACL 1 $\mu$g ml$^{-1}$ (**d**) (Cytofluorograf analysis).

that ACL accumulates unabated in these cells. The Pgp-modifying agents used in this study did not influence the cytotoxicity, accumulation or efflux of ACL. On the other hand, treatment of HB8065/R cells with SDZ PSC 833 increased the accumulation and decreased the efflux of EPI, DOX and DNR, resulting in multiplied sensitivity, which became equal to that of the parental HB8065/S cells. Thus, Pgp seemed to be the key determinant to explain the difference in cytotoxicity and cellular pharmacokinetics seen in the four anthracyclines. Our findings strongly indicate that ACL is a poor substrate for active outward transport by Pgp, which is in agreement with previous reports of MDR circumvention with 9-alkyl or morpholinyl substituted anthracyclines in cell lines (Scott *et al.*, 1986; Streeter *et al.*, 1986; Coley *et al.*, 1990). Two recent reports suggest that ACL may also circumvent drug resistance due to altered expression of topoisomerase II and glutathione S-transferase (Jensen *et al.*, 1993; Okuyama *et al.*, 1994).

The assessment of cytotoxicity was performed using growth inhibition assays that measure cell growth by electronic cell counting. Previous attempts to perform colony-forming assays in HB8065/R and HB8065/S cells have been unsuccessful because both lines failed to clone reproducibly on soft agar (Hall *et al.*, 1991). Although electronic assessment of growth inhibition does not discriminate between cells of poor or of good proliferating capacity, the results from growth inhibition assays of anthracycline toxicity have been shown to correlate with those of clonogenic assays in certain pairs of parental and MDR cell lines (Bhalla *et al.*, 1985; Hall *et al.*, 1991). Following a 3 h treatment course the growth inhibition assay revealed a 20-fold resistance to EPI, DOX and DNR in the resistant human hepatoma cell line relative to the parental line, which corresponded with approximately 2-fold lower intracellular drug accumulation in the resistant cells than in the parental cells. By extending the treatment duration to 3 days, the resistance to EPI and DOX was doubled and the resistance to DNR was halved. Thus, the dose–response relationship was schedule dependent. Correspondingly, it has previously been demonstrated that cell killing by doxorubicin is an exponential function of drug exposure time in Chinese hamster ovary cells (Bates *et al.*, 1985), and that lengthening the drug exposure time reduces the relative resistance to vincristine in two human colon carcinoma cell lines (Bates *et al.*, 1994).

Using flow cytometry, we demonstrated rapid accumulation of all the anthracyclines in both cell types within minutes of incubation, which is in agreement with previous *in vitro* and *in vivo* findings of rapid influxes of anthracyclines across cellular membranes (Meriwether & Bachur, 1972; Egorin *et al.*, 1974; Bachur, 1976). In both hepatoma cell lines, the fluorescence of DNR and ACL reached a plateau within 15 min. It was previously shown that ACL accumulates more rapidly than DNR or DOX in L1210 ascitic cells (Zenebergh *et al.*, 1982), and that the rate of cellular uptake increases by increasing lipophilicity of the anthracyclines (Skovsgaard, 1987; Wheeler and Kessel, 1980). EPI and DOX reached a plateau within 15 min in the resistant cells but continued to accumulate for 45–150 min in the parental cells. Thus, the drug accumulation was not only governed by physicochemical characteristics of the drugs, but also by cellular characteristics.

The confocal laser microscopic images confirmed the flow cytometric findings by showing less fluorescent brightness of EPI, DOX and DNR in the resistant cells than in the sensitive ones, and by showing no difference in fluorescent brightness of ACL in the two cell types. It has been demonstrated by others that the fluorescence intensity of intracellular DNR parallels measurements of uptake of the corresponding radioactive drug (Bhalla *et al.*, 1985). However, the intracellular anthracycline fluorescence does not accurately reflect the drug content because binding to DNA may cause quenching of the fluorescence, which is particularly pronounced for ACL (Skovsgaard, 1987;

**Figure 8** Changes in the intracellular drug fluorescence HB8065/R cells during 180 min of incubation with EPI (**a**), DOX (**b**), DNR (**c**) and ACL (**d**). The solid line represents cells treated with anthracycline alone (□), and the dashed line represents anthracycline-treated cells modified with either SDZ PSC 833 (●) or verapamil (▲). The time points for addition of anthracyclines and modifiers are indicated by open and closed arrows respectively, (FACScan analysis).

**Table I** Modification of drug efflux by SDZ PSC 833 in HB8065/R and HB8065/S cells. The modified drug efflux was compared with the natural efflux by means of independent samples *t*-test

| | *Drug efflux ($ng\ ml^{-1}$) (No modifier)* | *Drug efflux ($ng\ ml^{-1}$) SDZ PSC 833* | P |
|---|---|---|---|
| HB8065/R | | | |
| EPI | 350±2 | 235±18 | 0.001 |
| DOX | 154±11 | 80±3 | 0.003 |
| DNR | 712±36 | 515±37 | 0.009 |
| ACL | 2554±80 | 2472±87 | NS |
| HB8065/S | | | |
| EPI | 183±12 | 173±8 | NS |
| DOX | 66±4 | 46±11 | NS |
| DNR | 719±35 | 682±20 | NS |
| ACL | 1065±72 | 1023±62 | NS |

NS, not significant.

Tarasiuk *et al.*, 1989). The fluorescence intensity of free ACL has been measured to be 200 times that of nucleus-bound drug (Millot *et al.*, 1989). In the cytoplasm of the HB8065/S cells, and to a much lesser extent in the resistant HB8065/R cells, the anthracycline fluorescence appeared in numerous cytoplasmic vesicles. Similar vesicles have been demonstrated in both MDR cell lines and corresponding parental lines (Weaver *et al.*, 1991), and may represent drug accumulation in lysosomes. It is possible that higher drug concentrations may activate non-specific adsorptive endocytosis, which has been demonstrated in anthracycline-resistant Ehrlich ascites tumour cell lines (Sehested *et al.*, 1987).

Our results showed that low accumulation of EPI and DOX paralleled high efflux of these cytotoxics in the pair of cell lines studied. Because SDZ PSC 833 significantly reduced the efflux of EPI, DOX and DNR from the resistant human hepatoma cells but not from the sensitive ones, it is likely that Pgp was mainly responsible for the variations in the efflux of these anthracyclines. The efflux of ACL was not altered by SDZ PSC 833 in any of the cell types, and the greater efflux of ACL from the resistant human hepatoma cells was obviously unrelated to Pgp, but correlated with an increased accumulation of ACL in the resistant cells. Interestingly, it has been reported that there is no difference in the efflux of ACL between parental and resistant lines of human small-cell lung cancer and mouse mammary tumour (Coley *et al.*, 1993). Thus, there may exist alternative transport mechanisms for ACL in certain cell types that may also express high levels of Pgp.

The applied anthracycline doses in this study were in the upper range or slightly above achievable plasma concentrations in patients after standard bolus i.v. injections (Martini *et al.*, 1984; Paul *et al.*, 1989). An i.v. bolus injection of 60–120 mg $m^{-2}$ ACL yields initially 2–3 nmol $l^{-1}$ (Egorin *et al.*,

1982), which is approximately half of the highest applied dose of ACL in this study. The verapamil dose that restored the net drug accumulation in HB8065/R cells to the level of HB8065/S cells or above was approximately 10 times a clinically relevant plasma concentration in cardiac patients (Frishman *et al.*, 1982). On the other hand, it has been shown in rats that verapamil concentrates 40-fold in liver tissue compared with plasma (Hamann *et al.*, 1983). The SDZ PSC 833 dose that we applied in the culture media was similar to tolerable plasma concentrations obtained with the drug in clinical trials (Sonneveld *et al.*, 1994) and thus this drug seems more clinically promising.

In the present study, a comparison of four different anthracyclines with respect to cytotoxicity, intracellular accumulation, cellular efflux and response to Pgp-modifying agents strongly indicates Pgp as the major resistance mechanism responsible for the difference that has been demonstrated between ACL and the type I anthracyclines EPI, DOX and DNR. Therefore, we may conclude that the 9-alkyl trisaccharide ACL appears to be a poor substrate of Pgp in cultured human hepatoma cells. Translation of this unique feature of ACL into clinical practice would mean that treatment of unresectable hepatocellular carcinoma may benefit from tailoring the chemotherapeutic regimens according to the Pgp status of the tumour.

**Acknowledgements**
The authors are grateful to Dr Henrik S Huitfeldt for valuable help with the laser confocal microscopy and to Karen Johanne Beckstrøm, Reidum Hauge and May Ellen Lauritsen for excellent technical assistance. This work was financially supported by Medinnova, the Norwegian Cancer Society and the Research Council of Norway.

### References

ADEN DP, FOGEL A, PLOTKIN S, DAMJANOV I AND KNOWLES BB. (1979). Controlled synthesis of HBsAg in a differentiated human liver carcinoma-derived cell line. *Nature*, **282**, 615–616.

BACHUR NR, STEELE M, MERIWETHER WD AND HILDEBRAND RC. (1976). Cellular pharmacodynamics of several anthracycline antibiotics. *J. Med. Chem.*, **19**, 651–654.

BAEKELANDT M, KRISTENSEN G, HOLM R, NESLAND J AND TROPÉ C. (1994). P-gp expression as a possible marker for response to cisplatinum/epirubicin as a first line treatment in advanced ovarian cancer. *Anti-Cancer Drugs*, **5** (suppl. 1), 59.

BATES DA, FUNG H AND MACKILLOP WJ. (1985). Adriamycin uptake, intracellular binding and cytotoxicity in Chinese hamster ovary cells. *Cancer Lett.*, **28**, 213–221.

BATES SE, ZAHN Z, DICKSTEIN B, LEE JS, SCALA S, FOJO AT, PAULL K AND WILSON W. (1994). Reversal of multidrug resistance. *J. Hematother.*, **3**, 219–223.

BATIST G, TULPULE A, SINHA BK, KATKI AG, MYERS CE AND COWAN KH. (1986). Overexpression of a novel anionic glutathione transferase in multidrug-resistant breast cancer cells. *J. Biol. Chem.*, **261**, 15544–15549.

BEPPU T, OHARA C, YAMAGUCHI Y, ICHIKARA T, YAMANAKA T, KATAFUCHI S, IKEI S, MORI K, FUKUSHIMA S, NAKANO M AND OGAWA M. (1991). A new approach to chemoembolization for unresectable carcinoma using aclarubicin microspheres in combination with cisplatin suspended in iodized oil. *Cancer*, **68**, 2555–2560.

BHALLA K, HINDENBURG A, TAUB RN AND GRANT S. (1985). Isolation and characterization of an anthracycline-resistnat human leukemic cell line. *Cancer Res.*, **45**, 3657–3662.

BOESCH D, GAVERIAUX C, JACHEZ B, POURTIER-MANZANEDO A, BOLLINGER P AND LOOR F. (1991). In vivo circumvention of P-glycoprotein-mediated multidrug resistance of tumor cells with SDZ PSC 833. *Cancer Res.*, **51**, 4226–4233.

BROCK I, HIPFNER DR, NIELSEN BS, JENSEN PB, DEELEY RG, COLE SP AND SEHESTED M. (1995). Sequential coexpression of the multidrug resistance genes MRP and mdr1 and their products in VP-16 (etoposide)-selected H69 small cell lung cancer cells. *Cancer Res.*, **55**, 459–462.

CAMPOS L, GUYOTAT D, ARCHIMBAUD E, CALMARD-ORIOL P, TSURUO T, TRONCY J, TREILLE D AND FIERE D. (1992). Clinical significance of multidrug resistance P-glycoprotein expression on acute non-lymphoid leukemia cells at diagnosis. *Blood*, **79**, 473–476.

CHAN HS, THORNER PS, HADDAD G AND LING V. (1990). Immunohistochemical detection of P-glycoprotein: prognostic correlation in soft tissue sarcoma of childhood. *J. Clin. Oncol.*, **8**, 689–704.

CHAN HS, HADDAD G, THORNER PS, DEBOER G, LIN YP, ONDRUSEK N AND LING V. (1991). P-glycoprotein expression as a predictor of the outcome of therapy for neuroblastoma. *N. Engl. J. Med.*, **325**, 1608–1614.

COLE SP, CHANDA ER, DICKE FP, GERLACH JH AND MIRSKI SEL. (1991). Non-P-glycoprotein-mediated resistance in a small cell lung cancer cell line: evidence for decreased susceptibility to drug-induced DNA damage and reduced levels of topoisomerase II. *Cancer Res.*, **51**, 3345–3352.

COLE SP, BHARDWAJ G, GERLACH JH, MACKIE JE, GRANT CE, ALMQUIST KC, STEWARD AJ, KURZ EU, DUNCAN AM AND DEELEY RG. (1992). Overexpression of a transporter gene in a multidrug-resistant human lung cancer cell line. *Science*, **258**, 1650–1654.

COLEY HM, TWENTYMAN PR AND WORKMAN P. (1989). Improved cellular accumulation is characteristic of anthracyclines which retain high activity in multidrug resistant cell lines, alone or in combination with verapamil or cyclcosporin A. *Biochem. Pharmacol.*, **38**, 4467–4471.

COLEY HM, TWENTYMAN PR AND WORKMAN P. (1990). 9-alkyl morpholinyl anthracyclines in the circumvention of multidrug resistance. *Eur. J. Cancer*, **26**, 665–667.

COLEY HM, TWENTYMAN PR AND WORKMAN P. (1993). The efflux of anthracyclines in multidrug-resistant cell lines. *Biochem. Pharmacol.*, **46**, 1317–1326.

CORNWELL MM, PASTAN I AND GOTTESMAN MM. (1987). Certain calcium channel blockers bind specifically to multidrug resistant KB carcinoma membrane vesicles and inhibit drug binding to P-glycoprotein. *J. Biol. Chem.*, **262**, 2166–2170.

DANTCHEV D, SLIOUSSARTCHOUK V, PAINTRAND M, HAYAT M, BOURUT M AND MATHÉ G. (1979). Electron microscopic studies of the heart and light macroscopic studies of the skin after treatment of golden hamsters with adriamycin, detorubicin, AD-32 and aclacinomycin. *Cancer Treat. Rep.*, **63**, 875–888.

EGORIN MJ, HILDEBRAND RC, CIMINO EF AND BACHUR NR. (1974). Cytofluorescence localization of adriamycin and daunorubicin. *Cancer Res.*, **34**, 2243–2245.

EGORIN MJ, VAN ECHO D, FOX BM, WHITACRE M AND BACHUR NR. (1982). Plasma kinetics of aclacinomycin A and its major metabolites in man. *Cancer Chemother. Pharmacol.*, **8**, 41–46.

EIJDEMS EW, DE HAAS M, TIMMERMANN AJ, VAN DER SCHANS GP, KAMST E, DE NOOIJ, ASTALDI RICOTTI CG, BORST P AND BAAS F. (1995). Reduced topoisomerase II activity in multidrug-resistant human non-small-cell lung cancer cell lines. *Br. J. Cancer*, **71**, 40–47.

ENDICOTT JA AND LING V. (1989). The biochemistry of P-glycoprotein mediated multidrug resistance. *Annu. Rev. Biochem.*, **58**, 137–171.

FALKSON G, MACINTYRE JM AND MOERTEL CG. (1984). Primary liver cancer. *Cancer*, **54**, 977–980.

FOXWELL BMJ, MACKIE A, LING V AND RYFFEL B. (1989). Identification of the multidrug resistance-related P-glycoprotein as a cyclosporine binding protein. *Mol. Pharm.*, **36**, 543–546.

FRICHE E, JENSEN PB AND NISSEN NI. (1992). Comparison of cyclosporin A and SDZ PSC833 as multidrug-resistance modulators in a daunorubicin-resistant Ehrlich ascites tumor. *Cancer Chemother. Pharmacol.*, **30**, 235–237.

FRISHMAN W, KIRSTEN E, KLEIN M, PINE M, JOHNSON SM, HILLIS LD, PACKER M AND KATES R. (1982). Clinical relevance of verapamil plasma levels in stable angina pectoris. *Am. J. Cardiol.*, **50**, 1180–1184.

HALL KS, ENDRESEN L, HUITFELDT HS AND RUGSTAD HE. (1991). Induction of in vitro resistance to 4′-epidoxorubicin and cis-dichlorodiamineplatinum in hepatoma cells. *Anticancer Res.*, **11**, 817–824.

HAMADA H AND TSURUO T. (1986). Functional role for the 170- to 180-kDa glycoprotein specific to drug-resistant tumor cells as revealed by monoclonal antibodies. *Proc. Natl Acad. Sci. USA*, **83,** 7785–7789.

HAMANN SR, TODD GD AND MCALLISTER SR JR. (1983). The pharmacology of verapamil V. Tissue distribution of verapamil and norverapamil in rat and dog. *Pharmacology*, **27,** 1–8.

HORI S, SHIRAI M, HIRANO S, OKI T, INUIT T, TSUKAGOSHI S, ISHIZUKA M, TAKEUCHI T AND UMEZAWA H. (1977). Antitumour activity of new anthracycline antibiotics, Aclacinomycin-A and its analogs and their toxicity. *Gann*, **68,** 685–690.

ICHIKARA T, SAKAMOTO K, MORI K AND AKAGI M. (1989). Transcatheter arterial chemoembolization therapy for hepatocellular carcinoma using polyactic acid microspheres containing aclarubicin hydrochloride. *Cancer Res.*, **49,** 4357–4362.

ITSUBO M, ISHIKAWA T, TODA G AND TANAKA M. (1994). Immunohistochemical study of expression and cellular localization of the multidrug resistance gene product P-glycoprotein in primary liver carcinoma. *Cancer*, **73,** 298–303.

JENSEN PB, SØRENSEN BS, SEHESTED M, DERMANT EJ, KJELDSEN E, FRICHE E AND HANSEN HH. (1993). Different modes of anthracycline interaction with topoisomerase II. Separate structures critical for DNA-cleavage, and for overcoming topoisomerase II-related drug resistance. *Biochem. Pharmacol.*, **45,** 2025–2035.

JULIANO RL AND LING V. (1976). A surface glycoprotein modulating drug permeability in Chinese hamster ovary cell mutants. *Biochim. Biophys. Acta*, **455,** 152–162.

KNOWLES BB, HOWE CC AND ADEN DP. (1980). Human hepatocellular carcinoma cell lines secrete the major plasma proteins and hepatitis B surface antigen. *Science*, **209,** 497–499.

LEHNE G, DEANGELIS P, CLAUSEN OPF, HALL KS, HUITFELDT HS AND RUGSTAD HE. (1994). Pharmacokinetics and cytotoxicity of Epirubicin (EPI) in drug resistant human hepatoma cells (HB8065). *Int. J. Oncol.*, **4,** 1229–1235.

LEHNE G, DEANGELIS P, CLAUSEN OPF, EGELAND T, TSUROU T AND RUGSTAD HE. (1995). Diversity of externally and internally binding anti-P-glycoprotein antibodies in flow cytometric analysis of multidrug resistant cell lines. *Cytometry*, **20,** 228–237.

LICHT T, PASTAN I, GOTTESMAN M AND HERRMANN F. (1994). P-glycoprotein-mediated multidrug resistance in normal and neoplastic hematopoietic cells. *Ann. Hematol.*, **69,** 159–171.

MACHOVER D, GASIABURN J, DELGADO M, GOLDSCHMIDT E, HULHOVEN R, MISSET JL, DE VASSAL F, TAPIERO H, RIBAUD P, SCHWARZENBERG L AND MATHÉ G. (1984). Phase I–II study of aclarubicin for treatment of acute myeloid leukemia. *Cancer Treat. Rep.*, **68,** 881–886.

MARIE J-P, ZITTOUN R AND SIKIC BI. (1991). Multidrug resistance (mdr1) gene expression in adult acute leukemias: correlations with treatment outcome and in vitro drug sensitivity. *Blood*, **78,** 586–592.

MARTINI A, MORO E, PACCIARINI MA, TAMASSIA V, NATALE N AND PIAZZA E. (1984). Cross-over study pharmacokinetics and haemotological toxicity of 4′-epi-doxorubicin in cancer patients. *Int. J. Clin. Pharm. Res.*, **4,** 231–238.

MERIWETHER WD AND BACHUR NR. (1972). Inhibition of DNA and RNA metabolism by daunorubicin and adriamycin in L1210 mouse leukaemia. *Cancer Res.*, **32,** 1137–1142.

MILLOT JM, RASOANAIVO TDW, MORJANI H AND MANFAIT M. (1989). Role of aclacinomycin-A-doxorubicin association in reversal of doxorubicin resistance in K562 tumour cells. *Br. J. Cancer*, **60,** 678–684.

MIMNAUGH EG, DUSRE L, ATWELL J AND MYERS CE. (1989). Differential oxygen radical susceptibility of adriamycin-sensitive and -resistant MCF-7 human breast tumour cells. *Cancer Res.*, **49,** 8–15.

MORTENSEN SA. (1987). Aclarubicin: preclinical and clinical data suggesting less chronic cardiotoxicity compared with conventional anthracyclines. *Eur. J. Haematol.*, **47,** 21–31.

MUGGIA FM AND GREEN MD. (1991). New anthracycline antibiotics. *Crit. Rev. Oncol. Hematol.*, **11,** 43–64.

MULDER HS, DEKKER H, PINEDO HM AND LANKELMA J. (1995). The P-glycoprotein-mediated relative decrease in cytosolic free drug concentration is similar for several anthracyclines with varying lipophilicity. *Biochem. Pharmacol.*, **50,** 967–974.

OKI T, TAKEUCHI T, OKA S AND UMEZAWA H. (1981). New antibiotic aclacinomycin-A: experimental studies and correlations with clinical trials. *Recent Results Cancer Res.*, **76,** 21–40.

OKUYAMA T, MAEHARA Y, ENDO K, BABA H, ADACHI Y, KUWANO M AND SUGIMACHI K. (1994). Expression of glutathione S-transferase-pi and sensitivity of human gastric cancer cells to cisplatin. *Cancer*, **74,** 1230–1236.

PAUL C, LILIEMARK J, TIDEFELDT U, GARTHON G AND PETERSON C. (1989). Pharmacokinetics of daunorubicin and doxorubicin in plasma and leukemic cells from patients with acute nonlymphoblastic leukemia. *Ther. Drug Monit.*, **11,** 140–148.

PEDERSEN-BJERGAARD J, BRINCKER H, ELLEGAARD J, DRIVSHOLM A, FREUND L, JENSEN KB, JENSEN MK AND NISSEN NI. (1984). Aclarubicin in the treatment of nonlymphocytic leukemia refractory to treatment with daunorubicin and cytarabin: a phase II trial. *Cancer Treat. Rep.*, **68,** 1233–1238.

SCAMBIA G, PANICI PB, CONTU G, DE VINCENZO R, FERRANDINA G, ISOLA G, MACCIO A AND MANCUSO S. (1994). Mechanisms and modulation of resistance to anthracyclines (review). *Int. J. Oncol.*, **4,** 951–959.

SCOTT CA, WESTMACOTT D, BROADHURST MJ, THOMAS GJ AND HALL MJ. (1986). Absence of cross-resistance to adriamycin in human and murine cell cultures. *Br. J. Cancer*, **53,** 595–600.

SEHESTED M, SKOVSGAARD T, VANDUERS B AND WINTHER-NIELSON H. (1987). Increase in nonspecific adsorptive endocytosis in anthracycline- and vinca alkaloid-resistant Ehrlich ascites in tumor cell lines. *J. Natl Cancer Inst.*, **78,** 171–177.

SKOVSGAARD T. (1987). Pharmacodynamic aspects of aclarubicin with special reference to daunorubicin and doxorubicin. *Eur. J. Haematol.*, **38** (suppl. 47), 7–14.

SONNEVELD P, MARIE JP, LOKHORST HM, NOOTER K AND SCHOESTER M. (1994). Clinical modulation of multidrug resistance in VAD-refractory multiple myeloma: studies with cyclosporin and SDZ PSC 833. *Anti-Cancer Drugs*, **5** (suppl. 1), 72.

STREETER DG, JOHL JS, GORDON GR AND PETERS JH. (1986). Uptake and retention of morpholinyl anthracyclines by adriamycin-sensitive and -resistant P833 cells. *Cancer Chemother. Pharmacol.*, **16,** 247–252.

TARASIUK J, FRÉZARD F, GARNIER-SUILLEROT A AND GATTEGNO L. (1989). Anthracycline incorporation in human lymphocytes. Kinetics of uptake and nuclear concentration. *Biochim. Biophys. Acta*, **1013,** 109–117.

TEETER LD, HSU H-C, CURLEY SA, TONG MJ AND KUO MT. (1993). Expression of multidrug resistance (P-glycoprotein) MDR1 and MDR2 genes in human hepatocellular carcinomas and liver metastases of cooling tumors. *Int. J. Oncol.*, **2,** 73–80.

WEAVER JL, PINE AS, ASZALOS A, SHOENLEIN PV, CURRIER SJ, PADMANABHAN R AND GOTTESMAN MM. (1991). Laser scanning and confocal microscopy of daunorubicin, doxorubicin, and rhodamine 123 in multidrug-resistant cells. *Exp. Cell. Res.*, **196,** 323–329.

WHEELER CS AND KESSEL D. (1980). Influx of anthracyclines by murine leukemia cells as a function of drug lipophilicity and membrane fluidity. *Pharmacologist*, **22,** 291.

YUEN AR AND SIKIC BI. (1994). Multidrug resistance in lymphomas. *J. Clin. Oncol.*, **12,** 2453–2459.

ZENEBERGH A, BAURAIN R AND TROUET A. (1982). Cellular pharmacokinetics of aclacinomycin A in cultured L1210 cells. Comparison with daunorubicin and doxorubicin. *Cancer Chemother. Pharmacol.*, **8,** 243–249.